# The Tissue Fibrinolytic System Contributes to the Induction of Macrophage Function and CCL3 during Bone Repair in Mice

**DOI:** 10.1371/journal.pone.0123982

**Published:** 2015-04-20

**Authors:** Naoyuki Kawao, Yukinori Tamura, Yoshitaka Horiuchi, Katsumi Okumoto, Masato Yano, Kiyotaka Okada, Osamu Matsuo, Hiroshi Kaji

**Affiliations:** 1 Department of Physiology and Regenerative Medicine, Kinki University Faculty of Medicine, Osakasayama, Japan; 2 Life Science Research Institute, Kinki University, Osaka, Japan; Université de Lyon - Université Jean Monnet, FRANCE

## Abstract

Macrophages play crucial roles in repair process of various tissues. However, the details in the role of macrophages during bone repair still remains unknown. Herein, we examined the contribution of the tissue fibrinolytic system to the macrophage functions in bone repair after femoral bone defect by using male mice deficient in plasminogen (*Plg* ^–/–^), urokinase-type plasminogen activator (*uPA* ^–/–^) or tissue-type plasminogen activator (*tPA* ^–/–^) genes and their wild-type littermates. Bone repair of the femur was delayed in *uPA* ^–/–^ mice until day 6, compared with wild-type (*uPA* ^+/+^) mice. Number of Osterix-positive cells and vessel formation were decreased in *uPA* ^–/–^ mice at the bone injury site on day 4, compared with those in *uPA* ^+/+^ mice. Number of macrophages and their phagocytosis at the bone injury site were reduced in *uPA* ^–/–^ and *Plg* ^–/–^, but not in *tPA* ^–/–^ mice on day 4. Although uPA or plasminogen deficiency did not affect the levels of cytokines, including TNF-α, IL-1β, IL-6, IL-4 and IFN-γ mRNA in the damaged femur, the elevation in CCL3 mRNA levels was suppressed in *uPA* ^–/–^ and *Plg* ^–/–^, but not in *tPA* ^–/–^ mice. Neutralization of CCL3 antagonized macrophage recruitment to the site of bone injury and delayed bone repair in *uPA* ^+/+^, but not in *uPA* ^–/–^ mice. Our results provide novel evidence that the tissue fibrinolytic system contributes to the induction of macrophage recruitment and CCL3 at the bone injury site, thereby, leading to the enhancement of the repair process.

## Introduction

Fractures are one of the most frequent injuries of the musculoskeletal system. Optimal treatment of fractures requires the knowledge of the process of bone repair [1]. The process of bone repair can be divided into three overlapping phases: inflammation, repair and remodeling [1]. Neutrophils are rapidly recruited to the site of bone injury [1]. Subsequently, macrophages are attracted to the injured site by various chemoattractants, including the CC chemokines CCL2, CCL3 and CCL4 [1–3]. Macrophages play a crucial role in the tissue repair process through the release of growth factors, engulfment of cellular debris and efferocytosis of apoptotic cells [2,4]. The activated macrophages release pro-angiogenic factors, which stimulate vessel formation at the damaged site. However, the details in the mechanism of macrophage function during bone repair still remain unknown.

Urokinase-type plasminogen activator (uPA) and tissue-type plasminogen activator (tPA) convert the inactive pro-enzyme plasminogen to the active serine protease plasmin, which is a pivotal component of the fibrinolytic system [5]. The fibrinolytic system plays many physiological and pathophysiological roles in addition to its proteolytic degradation of fibrin clots. The fibrinolytic system activates the tissue proteolytic system and modulates the release of growth factors such as vascular endothelial growth factor (VEGF) through the degradation of the extracellular matrix [6,7]. Previous studies indicate that the tissue fibrinolytic system is involved in the tissue-repair process of the skin and liver [8–10].

Plasminogen activators, tPA and uPA, are released from osteoblasts and osteoclasts by stimuli with cytokines and hormones such as parathyroid hormone [11–14]. Daci *et al*. reported that bone mass is increased in uPA and tPA double-deficient mice [15]. Deficiency in PA inhibitor-1 (PAI-1), an endogenous negative regulator of uPA and tPA, partially protects against bone loss in estrogen-deficient mice and diabetic female mice [16,17]. These findings suggest that the tissue fibrinolytic system regulates bone metabolism under physiological and pathological conditions. We recently demonstrated that plasminogen is crucial for bone repair using plasminogen-deficient mice [18]. Moreover, PAI-1 is involved in impaired bone repair associated with diabetes in female mice [19]. Taken together, our findings indicate that the tissue fibrinolytic system is essential for the process of bone repair. Evidence suggests that tissue macrophages are related to the tissue fibrinolytic system during the tissue repair process [10,18,20]. In our previous study, plasminogen deficiency suppressed the accumulation of macrophages and the expression of transforming growth factor-β (TGF-β) and VEGF at the damaged site after a bone defect, leading to an impaired bone repair process in mice [18]. Although these findings suggest that macrophages might play some roles in the tissue fibrinolytic system during the bone repair process, the detailed mechanisms remain unknown.

We previously revealed that the accumulation of macrophages was impaired by plasminogen deficiency on day 4 after femoral bone defect in mice [18], suggesting that macrophages are important for the tissue-fibrinolytic system-related bone repair process at earlier time points. In the present study, we therefore investigated the contribution of macrophage for the tissue fibrinolytic system-related bone repair process after femoral bone defect using mice with plasminogen, uPA or tPA deficiency and their wild-type littermates especially at earlier time points.

## Materials and Methods

### Materials

Anti-Osterix (# ab22552), anti-CD31 (# ab28364), anti-VEGF (# ab46154) and anti-TGF-β (# ab66043) antibodies were obtained from Abcam (Cambridge, UK). Anti-alkaline phosphatase (ALP, # PAB12279), β-actin (# 4967) and anti-F4/80 (# MCA497R) antibodies were purchased from Abnova (Taipei, Taiwan), Cell Signaling Technology (Danvers, MA, USA) and AbD Serotec (Raleigh, NC, USA), respectively. Anti-bone morphogenetic protein (BMP)-2 (# sc-6895) and anti-hypoxia-inducible factor (HIF)-1α (# sc-10790) antibodies were purchased from Santa Cruz Biotechnology (Santa Cruz, CA, USA). A neutralizing goat anti-CCL3/macrophage inflammatory protein-1α antibody (# AB-450-NA) and normal goat IgG were obtained from R&D Systems (Minneapolis, MN, USA).

### Ethics statement

This study was performed in strict accordance with the institutional guidelines for the use and care of laboratory animals at Kinki University. The study protocol was approved by the Experimental Animal Welfare Committee of Kinki University (permit number: KAME-23-023). All surgery was performed under isoflurane anesthesia and all efforts were made to minimize suffering.

### Animals

Male mice with plasminogen (*Plg* ^-/-^), uPA (*uPA* ^-/-^) or tPA (*tPA* ^-/-^) gene deficiency and their wild-type littermates (*uPA* ^+/+^, *Plg* ^+/+^ and *tPA* ^+/+^, respectively) obtained from heterozygous breeding pairs were used for the present study. All genotypes of the mice used for the present study were determined by polymerase chain reaction (PCR) analysis of tail DNA. The genetic background of all mice was mixed C57BL/6J (75%) and 129/SvJ (25%) [21–23]. To minimize the effects of mouse strain differences, age-matched and weight-matched male mice, 8-weeks-old and each weighing 22–26 g, were used.

### Bone defect model

A bone defect was induced in the mice according to the method as described previously [18,19]. Briefly, the mice were anesthetized using 2% isoflurane and the surface of the right femoral bone was exposed. A hole was made in the diaphysis of the right femoral bone in the proximal region, 4 mm from the knee joint, using a drill with a diameter of 0.9 mm.

For the anti-CCL3 antibody treatment experiments, the neutralizing goat anti-CCL3 antibody and normal goat IgG were dissolved in sterile physiological saline at 1 mg/mL. *uPA* ^+/+^ and *uPA* ^-/-^ mice were administered twice intraperitoneal doses of 100 μg/mouse of the neutralizing goat anti-CCL3 antibody or normal goat IgG at immediately after the femoral bone defect and on day 2.

### *In vivo* quantitative computed tomography (qCT) analysis

The *in vivo* qCT analysis was performed according to our previous studies [18,19] and the guidelines of the American Society for Bone and Mineral Research [24]. The femur of mice was scanned using an X-ray CT system (Latheta LCT-200; Hitachi Aloka Medical, Tokyo, Japan) under 2% isoflurane anesthesia. CT images were acquired using the following parameters: 50 kVp tube voltage, 500 μA tube current, 3.6 ms integration time, 48 mm axial field of view and 48 μm isotropic voxel size [18,19]. Volume-rendered 3-dimensional CT images were reconstructed using VGStudio MAX2.2 (Nihon Visual Science, Tokyo, Japan) for quantification of the area of bone defect. The drill hole was visualized as a white circle on day 1 after surgery. The white region in the drill hole was quantified using ImageJ (http://rsbweb.nih.gov/ij/download.html), as the area of the bone defect.

### Histological analysis

The mice were euthanized with pentobarbital sodium (over 50 mg/kg, intraperitoneally) on days 4 and 7 after surgery. The damaged femur was removed and embedded in paraffin after decalcification with 22.5% formic acid and 340 mM sodium citrate solution. Four-micrometer-thick sections were obtained from the damaged site of femur. The sections were stained with hematoxylin/eosin (HE). Immunostaining for Osterix, ALP, CD31 or F4/80 was performed as described previously [18,25,26]. Briefly, the sections including the damaged site were incubated with rabbit polyclonal anti-Osterix antibody at a dilution of 1:200, rabbit polyclonal anti-ALP antibody at a dilution of 1:100, rabbit polyclonal anti-CD31 antibody at a dilution of 1:100 or rat monoclonal anti-F4/80 antibody at a dilution of 1:1000, followed by incubation with Histofine simple stain mouse MAX-PO (rabbit) or Histofine simple stain mouse MAX-PO (rat) (Nichirei Biosciences, Tokyo Japan). Tyramide signal amplification (TSA) system (PerkinElmer, Waltham, MS, USA) was used for the detection of positive signals. These sections were counterstained with diamino phenylindole. The numbers of Osterix-positive, ALP-positive and F4/80-positive cells per 0.1 mm^2^ of the microscopic fields were blindly quantified as described previously [18]. The total luminal area and the number of CD31-immunopositive blood vessels per 0.1 mm^2^ of the microscopic fields were quantified using ImageJ in a blinded manner as described previously [18]. The sections were stained with tartrate-resistant acid phosphatase (TRAP) to quantify osteoclasts [18]. The number of TRAP-positive multinucleated cells (MNCs) on the bone surface per 1 mm was quantified in a blinded manner. The regions of interest used for quantification of Osterix, ALP, TRAP and F4/80-positive cells as well as CD31-positive blood vessels were defined between the edges of damaged cortical bone without bone marrow cells in the sections.

The sections were stained with Alcian blue and toluidine blue to determine cartilage formation [18]. The area of cartilage matrices, including proteoglycans and glycosaminoglycans, were blindly quantified by measuring the Alcian blue-positive area and the metachromatic area in the sections stained with toluidine blue using Mac SCOPE (Mitani Co., Fukui, Japan) as described previously [18]. The regions of interest used for quantification of area of cartilage matrices were defined on the cortical bone at the damaged site. Three sections obtained from each femur were used for the quantitative analysis.

### Transmission electron microscopy

The transmission electron microscopy analysis was performed as previously described [10]. Under pentobarbital sodium (50 mg/kg) anesthesia, the mice were perfused transcardially with 50 mL of physiological saline and, subsequently, with 50 mL of 2.5% glutaraldehyde (Nisshin EM, Tokyo, Japan) in phosphate buffer (pH 7.4) on day 4 after femoral bone defect. The femur was removed, demineralized in 22.5% formic acid and 340 mM sodium citrate solution, cut into small pieces (1 × 3 × 5 mm), and post-fixed in the same fixative overnight at 4°C. The small pieces were further fixed in 1% buffered osmium tetroxide (Nisshin EM, Tokyo, Japan) for 1 h at 4°C and pre-stained with 0.5% uranyl acetate for 1 h at 4°C. After dehydration in an ethanol series, the pieces were embedded in an epoxy resin and 70-nm-thick sections were obtained from the damaged site on the femur. The ultra-thin sections were stained with 3% uranyl acetate for 20 min at room temperature. The stained sections were photographed under an electron microscope (HT-7700; Hitachi High-Technologies Co., Tokyo, Japan) at an accelerating voltage of 100 kV.

The ratio of macrophages in which erythrocytes were observed is indicated as the ratio of these macrophages to, at least, 25 macrophages at the damaged site of the femur in each mouse in a blinded manner. Macrophages in which the nucleus was not observed were excluded from the analysis.

### Western blot analysis

A damaged femur and intact femur from the contralateral side were homogenized in a tissue lysis buffer (Cell Signaling Technology) supplemented with protease inhibitors. Western blot analysis was performed as described previously [18]. The bicinchoninic acid assay was performed for quantification of total protein levels. After separation of the protein aliquots by SDS-PAGE, the proteins were transferred to polyvinylidene fluoride membrane. The membrane was blocked by a solution containing 20 mM Tris-HCl (pH 7.6), 137 mM NaCl, 0.1% Tween 20 and 3% skim milk. The membrane was incubated with primary antibodies, followed by incubation with the appropriate secondary antibody. The bands were visualized by the ECL select detection system (GE Healthcare, Tokyo, Japan).

### Quantitative real-time PCR

Total RNA was isolated from the tissues and cells using an RNeasy Mini Kit (Qiagen, Hilden, Germany). The incorporation of SYBR Green into double-stranded DNA was assessed by quantitative real-time PCR using an ABI StepOne Real-Time PCR System (Applied Biosystems, Carlsbad, CA, USA) as described previously [18]. The primers for real-time PCR are shown in S1 Table. The mRNA levels of the target genes were normalized with the glyceraldehyde-3-phosphate dehydrogenase mRNA levels.

### Preparation of primary osteoblasts

Calvarial osteoblasts were obtained from *uPA* ^+/+^ and *uPA* ^-/-^ mice according to the method as described previously [18]. Briefly, the calvaria from 3-day-old mice were digested four times with Minimum Essential Medium Alpha Modification (α-MEM) containing 1 mg/mL collagenase and 0.25% trypsin for 20 min at 37°C with gentle agitation. The cells from the second, third and fourth digestions were collected and cultured in α-MEM with 10% fetal bovine serum (FBS) and 1% penicillin-streptomycin. The medium was changed twice a week.

### ALP activity

ALP activity in primary osteoblasts was analyzed, as described previously [27]. After reaching confluence, primary osteoblasts, in a 24-well culture plate, were washed three times with phosphate buffered saline and sonicated in 600 μL of distilled water. The ALP activity was quantified using a LabAssay ALP kit (Wako Pure Chem., Osaka, Japan), according to the manufacturer's instructions. The absorbance was measured at 405 nm and each value was normalized to the total protein content.

### Mineralization assay

Mineralization of primary osteoblasts was analyzed by Alizarin Red staining as described previously [18,28]. After reaching confluence, the primary osteoblasts were cultured in α-MEM with 10% FBS, 10 mM β-glycerophosphate and 50 μg/mL ascorbic acid for 3 weeks. The cells were stained with Alizarin Red S solution to detect calcification. The stained cells were destained with 10% cetylpyridinium chloride, after which the absorbance of the extracted stain was measured at 570 nm.

### Statistical analysis

Data are represented as the mean ± standard error of the mean (SEM). Statistical analyses were performed by Mann-Whitney *U* test for comparisons of 2-group. Two-way ANOVA followed by Tukey’s test was performed for multiple comparisons when data presented a normal distribution, assessed by Kolmogorov-Smirnov test, and equal variances, assessed by Bartlett's test. Steel-Dwass test was performed when data did not present a normal distribution or equal variances for multiple comparisons. Differences among experimental groups were considered significant when *P* values were less than 0.05.

## Results

### Delayed bone repair at earlier time points in *uPA* ^-/-^ mice

**Fig 1:**
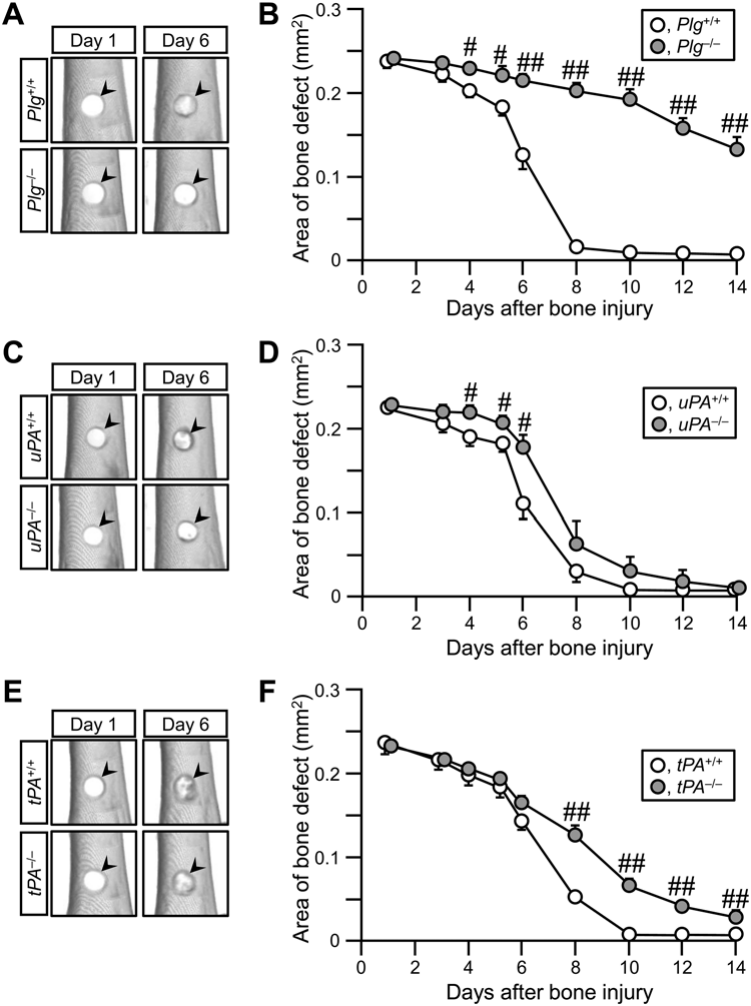
Delayed bone repair in *Plg* ^-/-^, *uPA* ^-/-^ and *tPA* ^-/-^ mice. (A, C, E) Quantitative computed tomography (qCT) images of the damaged site of femurs in *Plg* ^**+/+**^ and *Plg* ^**-/-**^ (A), *uPA* ^**+/+**^ and *uPA* ^**-/-**^ (C) as well as *tPA* ^**+/+**^ and *tPA* ^**-/-**^ mice (E). The arrowheads indicate the site of bone defect. (B, D, F) Quantification of the bone defect area as assessed by qCT in *Plg* ^**+/+**^ and *Plg* ^**-/-**^ (B), *uPA* ^**+/+**^ and *uPA* ^**-/-**^ (D) as well as *tPA* ^**+/+**^ and *tPA* ^**-/-**^ mice (F). The data represent the mean ± SEM from 8 (B, F) and 6 (D) mice. ^**##**^ *P* < 0.01 and ^**#**^ *P* < 0.05 (Mann-Whitney *U* test).

Bone repair was delayed in *Plg* ^-/-^ mice from day 4 to day 14 (Fig 1A and 1B) and in *tPA* ^-/-^ mice from day 8 to day 14 (Fig 1E and 1F), compared with that in their wild-type mice. The data in *Plg* ^-/-^ and *tPA* ^-/-^ mice were compatible with those of our previous studies [18,29]. In *uPA* ^-/-^ mice, bone repair was significantly delayed from day 4 to day 6, but not from day 8 to 14, compared with *uPA* ^+/+^ mice (Fig 1C and 1D). In the HE-stained sections, the newly generated bone tissues were similarly observed in *uPA* ^+/+^ and *uPA* ^-/-^ mice on day 7 (S1 Fig). These results suggest that uPA contributes to the bone repair process at earlier time points.

### Effects of uPA deficiency on the numbers of osteoblasts and osteoclasts, decrease in the area of bone defect and formation of cartilage at the damaged site

**Fig 2:**
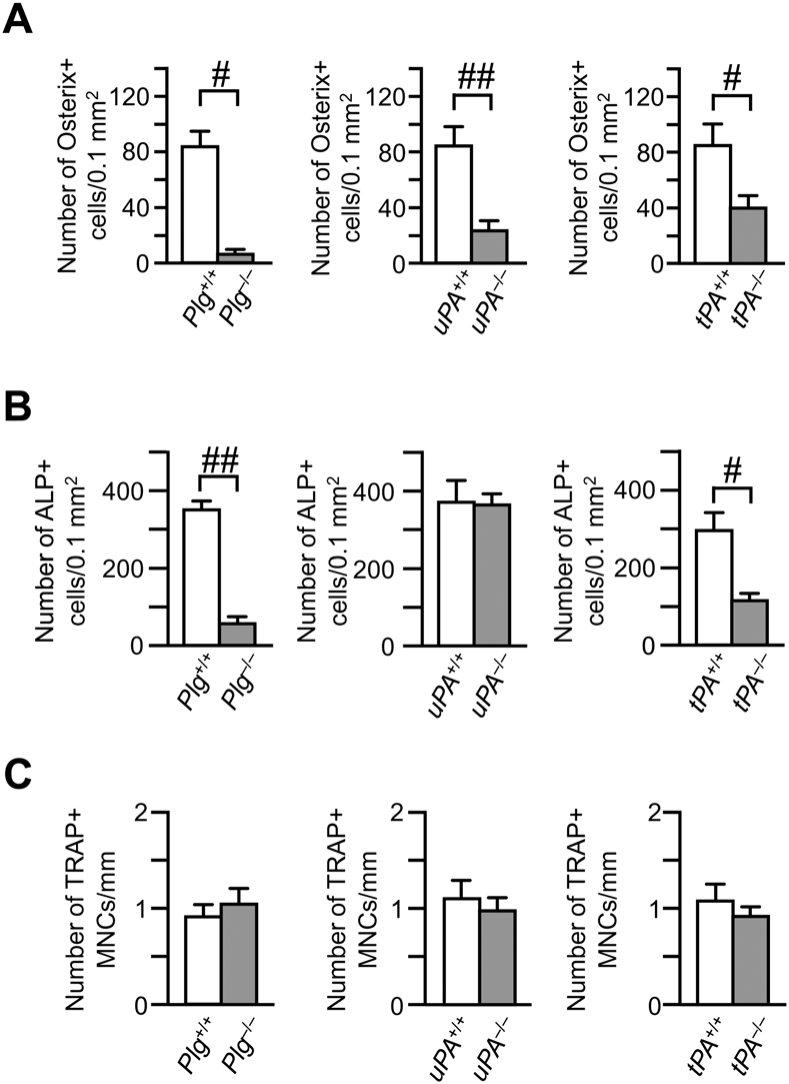
Decrease in the number of Osterix-positive cells at the damaged site on day 4 after femoral bone defect in *Plg* ^-/-^, *uPA* ^-/-^ and *tPA* ^-/-^ mice. (A) Quantification of Osterix-positive cells per 0.1 mm^**2**^ of the microscopic fields at the damaged site on day 4 in *Plg* ^**-/-**^, *uPA* ^**-/-**^, *tPA* ^**-/-**^ and each wild-type mice. (B) The number of alkaline phosphatase (ALP)-positive cells per 0.1 mm^**2**^ of the microscopic fields in the damaged site on day 7 in *Plg* ^**-/-**^, *uPA* ^**-/-**^, *tPA* ^**-/-**^ and each wild-type mice. (C) The number of tartrate-resistant acid phosphatase (TRAP)-positive multinucleated cells (MNCs) per 1 mm of bone surface at the damaged site on day 7 in *Plg* ^**-/-**^, *uPA* ^**-/-**^, *tPA* ^**-/-**^ and each wild-type mice. The data represent the mean ± SEM from 4–5 (A) and 5–7 (B, C) mice. ^**##**^ *P* < 0.01 and ^**#**^ *P* < 0.05 (Mann-Whitney *U* test).

We next examined osteoblasts at the damaged site after femoral bone defect at earlier time point to investigate the influence of macrophage-related bone repair process. The number of Osterix-positive cells in *Plg* ^-/-^, *uPA* ^-/-^ and *tPA* ^-/-^ mice were significantly decreased on day 4, compared with those in *Plg* ^+/+^, *uPA* ^+/+^ and *tPA* ^+/+^ mice, respectively (Fig 2A). The data in *Plg* ^-/-^ and *tPA* ^-/-^ mice were compatible with our previous studies [18,29]. The numbers of ALP-positive cells, which represent osteoblasts, were decreased at the damaged site in *Plg* ^-/-^ and *tPA* ^-/-^ mice on day 7, compared with that in their wild-type mice (Fig 2B). On the other hand, the number of ALP-positive cells was not significantly different between *uPA* ^+/+^ and *uPA* ^-/-^ mice on day 7 (Fig 2B). The numbers of TRAP-positive MNCs, which represent osteoclasts, were not significantly different between *Plg* ^+/+^ and *Plg* ^-/-^ mice, *uPA* ^+/+^ and *uPA* ^-/-^ mice or *tPA* ^+/+^ and *tPA* ^-/-^ mice on day 7 (Fig 2C).

There was no significant difference in the cartilage matrix between *uPA* ^+/+^ and *uPA* ^-/-^ mice in sections stained with Alcian and toluidine blue on day 7 after femoral bone defect (S2 Fig). The levels of aggrecan, and types II and X collagen mRNA were similar in the damaged femurs between *uPA* ^+/+^ and *uPA* ^-/-^ mice (S2 Fig).

The levels of Runx2, Osterix, ALP and type I collagen mRNA were comparable between primary osteoblasts from *uPA* ^+/+^ and *uPA* ^-/-^ mice (S3 Fig). Moreover, ALP activity and the mineralization of primary osteoblasts were comparable between *uPA* ^+/+^ and *uPA* ^-/-^ mice (S3 Fig).

### Impaired vessel formation at the damaged site on day 4 after femoral bone defect in *Plg* ^-/-^, *uPA* ^-/-^ and *tPA* ^-/-^ mice

**Fig 3:**
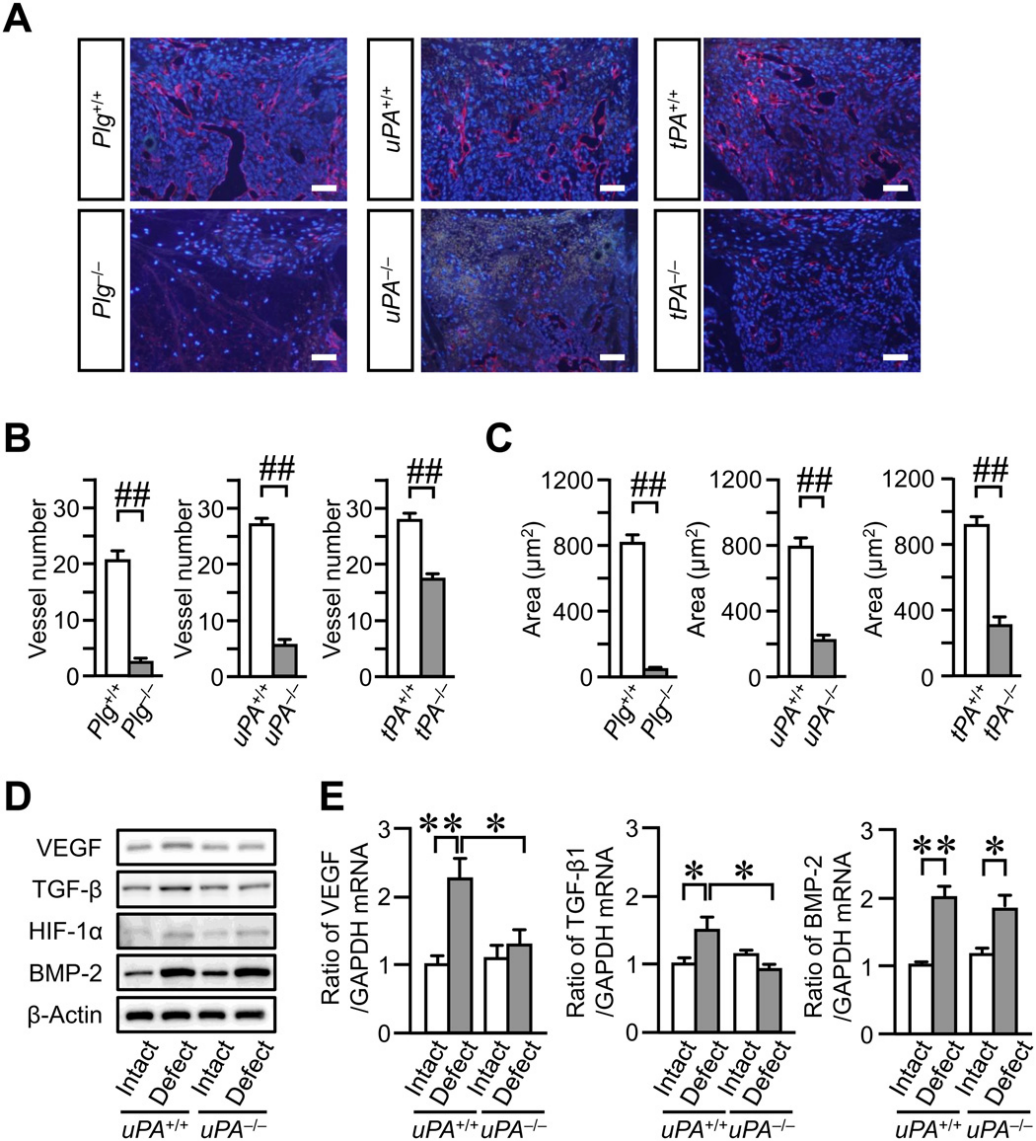
Impaired vessel formation at the damaged site after femoral bone defect in *Plg* ^-/-^, *uPA* ^-/-^ and *tPA* ^-/-^ mice. (A) Photographs of CD31-positive blood vessels at the damaged site on day 4 after femoral bone defect. Scale bars indicate 50 μm. (B, C) Quantification of blood vessels at the damaged site on day 4 after femoral bone defect. The number (B) and total luminal area (C) of CD31-positive blood vessels per 0.1 mm^**2**^ of the microscopic fields in the damaged site. The data represent the mean ± SEM of 5 mice. (D) Western blot analysis of VEGF, TGF-β, HIF-1α and BMP-2 levels in the damaged and contralateral intact femurs on day 4 after a bone defect in *uPA* ^**+/+**^ and *uPA* ^**-/-**^ mice. The results represent experiments performed on 5 mice in each group. (E) Relative levels of VEGF, TGF-β and BMP-2 mRNA in the damaged and contralateral intact femurs on day 4 after a bone defect in *uPA* ^**+/+**^ and *uPA* ^**-/-**^ mice. The data are expressed relative to glyceraldehyde-3-phosphate dehydrogenase (GAPDH) mRNA values. The data represent the mean ± SEM of 6 mice. ^**##**^ *P* < 0.01 (Mann-Whitney *U* test). **P < 0.01 and *P < 0.05 (Tukey’s test).

Since vessel formation is a critical event in bone repair, we examined the effects of plasminogen, uPA and tPA deficiency on vessel formation. The number and luminal areas of vessels at the damaged site in *Plg* ^-/-^, *uPA* ^-/-^ and *tPA* ^-/-^ mice were significantly decreased on day 4 after femoral bone defect, compared with those in *Plg* ^+/+^, *uPA* ^+/+^ and *tPA* ^+/+^ mice, respectively (Fig 3A–3C). On the other hand, vessel formation was comparable between *uPA* ^+/+^ and *uPA* ^-/-^ mice on day 7 (S4 Fig). VEGF plays an important role in vessel formation. The expression of VEGF is regulated by several growth factors, such as TGF-β, BMP-2 and HIF-1α at the damaged site after bone injury [30–32]. We therefore examined the levels of these factors in the damaged femur of *uPA* ^+/+^ and *uPA* ^-/-^ mice on day 4. The levels of VEGF and TGF-β in the damaged femurs were lower in *uPA* ^-/-^ mice compared with those in *uPA* ^+/+^ mice (Fig 3D and 3E). In contrast, the levels of HIF-1α and BMP-2 in the damaged femur were similar between *PA* ^+/+^ and *uPA* ^-/-^ mice (Fig 3D and 3E).

### Decrease in the number of macrophages and ratio of macrophage phagocytosis at the damaged site in *Plg* ^-/-^ and *uPA* ^-/-^, but not in *tPA* ^-/-^ mice

**Fig 4:**
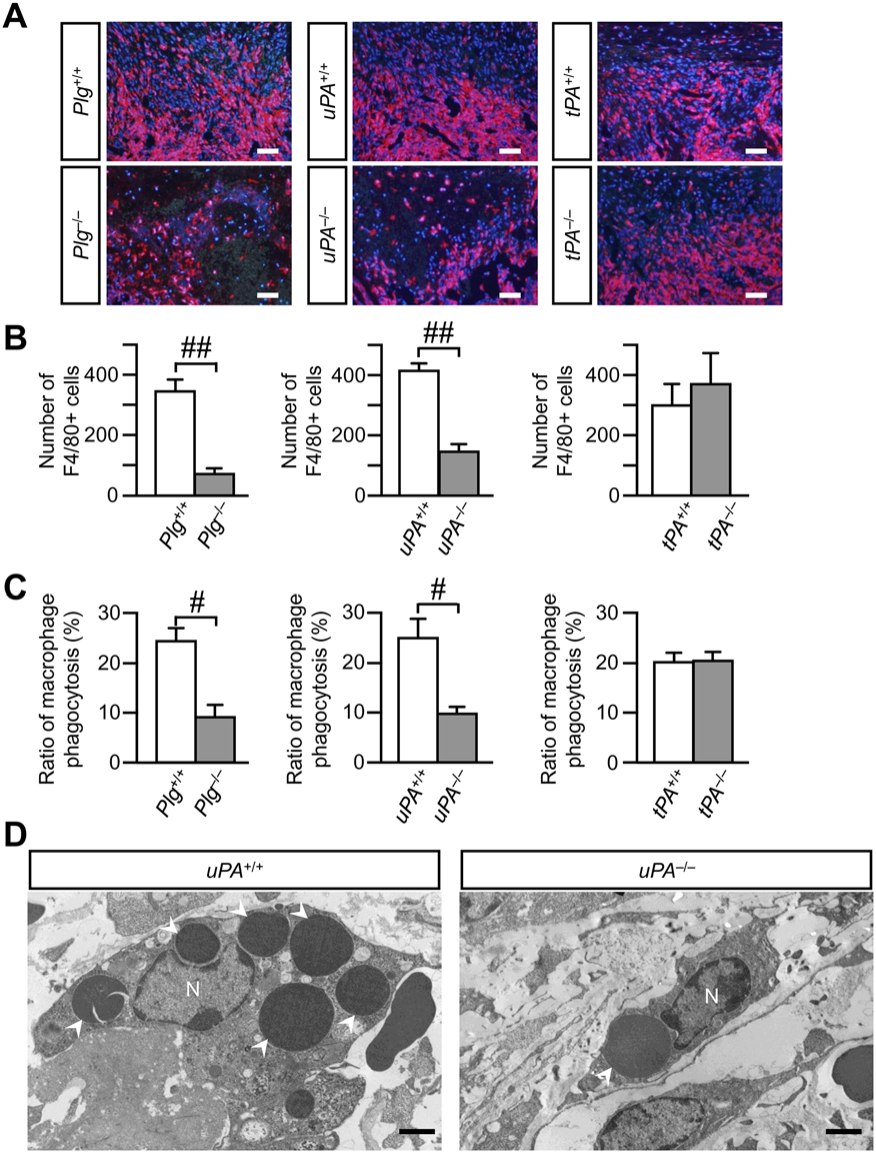
Decrease in the number of macrophages and ratio of macrophage phagocytosis at the damaged site on day 4 after femoral bone defect in *Plg* ^-/-^and *uPA* ^-/-^, but not in *tPA* ^-/-^ mice. (A) Photographs of F4/80-positive cells at the damaged site on day 4 after femoral bone defect. Scale bars indicate 50 μm. (B) Quantification of F4/80-positive cells at the damaged site on day 4 after femoral bone defect. The data represent the mean ± SEM of 5 mice. (C) The ratio of macrophage phagocytosis at the damaged site on day 4 assessed by transmission electron microscopy. The data represent the mean ± SEM of 4 mice. (D) Transmission electron microscopic photographs of macrophages at the damaged site on day 4 in *uPA* ^**+/+**^ and *uPA* ^**-/-**^ mice. The results represent experiments performed on 4 mice in each group. Arrowheads indicate erythrocytes in macrophages. Scale bars indicate 2 μm. N: nucleus. ^**##**^ *P* < 0.01 and ^**#**^ *P* < 0.05 (Mann-Whitney *U* test).

Since recruited macrophages are thought to be one of the major sources of angiogenic factors, including TGF-β, during tissue repair, we examined the accumulation of macrophages to the damaged site of the femur on day 4. The number of macrophages was counted as the number of F4/80-positive cells. The numbers of macrophages in *Plg* ^-/-^ and *uPA* ^-/-^ mice were significantly decreased at the damaged site of the femur on day 4, compared with those in *Plg* ^+/+^ and *uPA* ^+/+^ mice, respectively (Fig 4A and 4B). However, no significant difference was observed in the number of macrophages at the damaged site between *tPA* ^+/+^ and *tPA* ^-/-^ mice (Fig 4A and 4B). Next, we examined the ultra-structural changes of the macrophages that were recruited to the damaged site using transmission electron microscopy. Recruited macrophages with well-extended pseudopodia at the damaged site were observed on day 4 in *uPA* ^+/+^ mice (Fig 4D). These macrophages engulfed erythrocytes in *uPA* ^+/+^ mice (Fig 4D). Conversely, recruited macrophages had less extended pseudopodia in *uPA* ^-/-^ mice, as compared with *uPA* ^+/+^ mice (Fig 4D). Phagocytosis of erythrocytes by macrophages at the damaged site was rarely observed in *Plg* ^-/-^ and *uPA* ^-/-^ mice compared with that in *Plg* ^+/+^ and *uPA* ^+/+^ mice, respectively (Fig 4C), although the ratio of macrophage phagocytosis was similar between *tPA* ^+/+^ and *tPA* ^-/-^ mice (Fig 4C).

### Levels of cytokines in the damaged femurs

**Fig 5:**
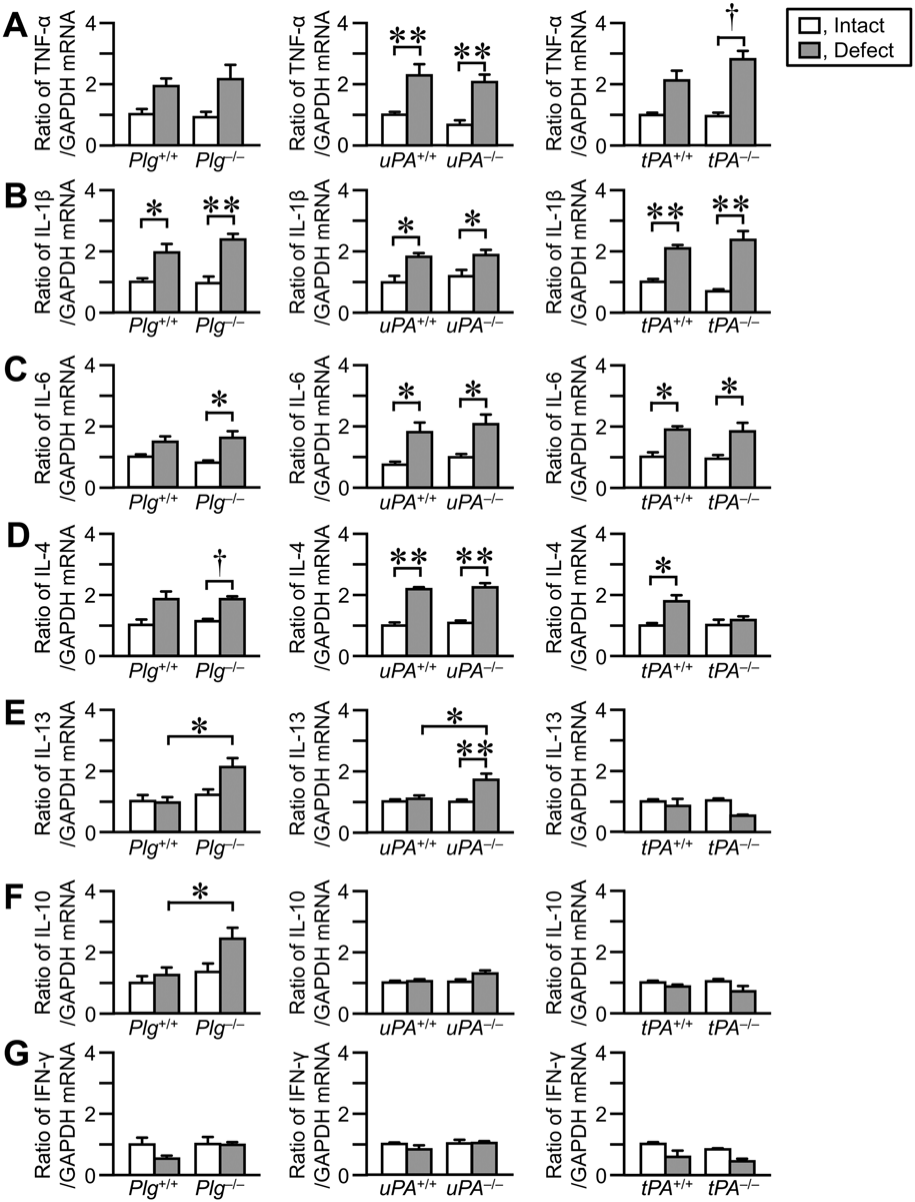
Effects of plasminogen, uPA or tPA deficiency on the levels of cytokines in the damaged and contralateral intact femurs on day 4. (A–G) Real time-PCR analysis of tumor necrosis factor (TNF)-α (A), interleukin (IL)-1β (B), IL-6 (C), IL-4 (D), IL-13 (E), IL-10 (F) and interferon (IFN)-γ (G) mRNA in the femurs on day 4. The data are expressed relative to GAPDH mRNA values. The data represent the mean ± SEM of 5–6 mice. **P < 0.01 and *P < 0.05 (Tukey’s test). ^**†**^ *P* < 0.05 (Steel-Dwass test).

We next examined the levels of cytokine mRNA in the femurs on day 4 after femoral bone defect. The levels of tumor necrosis factor (TNF)-α, interleukin (IL)-1β, IL-6 and IL-4 mRNA seemed to be elevated in the damaged femurs, compared with those in the contralateral intact femurs in *Plg* ^+/+^, *uPA* ^+/+^ and *tPA* ^+/+^ mice (Fig 5A–5D). The levels of IL-13, IL-10 and interferon (IFN)-γ mRNA were comparable between the damaged femurs and contralateral intact femurs in *Plg* ^+/+^, *uPA* ^+/+^ and *tPA* ^+/+^ mice (Fig 5E–5G). Plasminogen, uPA or tPA deficiency did not affect the levels of TNF-α, IL-1β, IL-6, IL-4 and IFN-γ mRNA in the damaged femurs (Fig 5A–5D and 5G). The levels of IL-13 and IL-10 mRNA were significantly elevated in the damaged femurs of *Plg* ^-/-^ or *uPA* ^-/-^ mice, compared with those in *Plg* ^+/+^ or *uPA* ^+/+^ mice, respectively (Fig 5E and 5F).

### Levels of CCL2, CCL3 and CCL4 mRNA in the damaged femur

**Fig 6:**
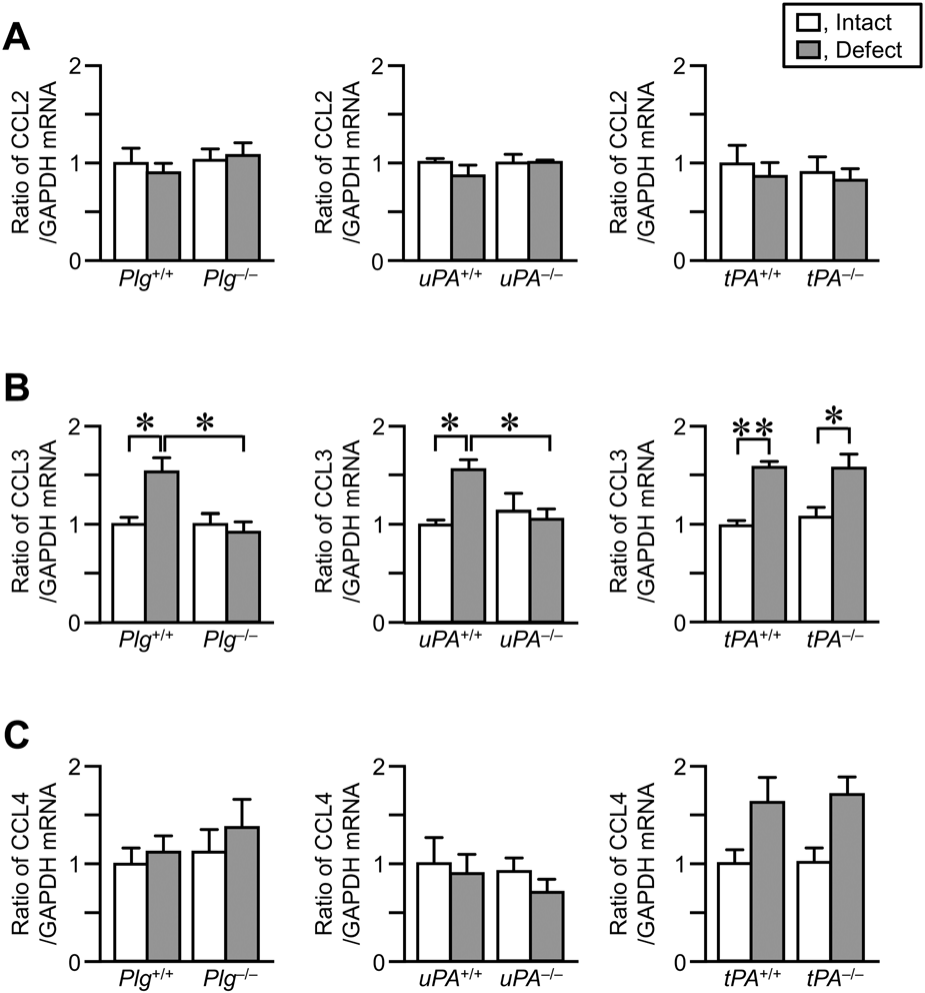
Effects of uPA, plasminogen or tPA deficiency on the levels of CCL2, CCL3 and CCL4 mRNA in the damaged and contralateral intact femurs on day 4. (A–C) Real time-PCR analyses of CCL2 (A), CCL3 (B) and CCL4 (C) mRNA in the femurs on day 4. The data are expressed relative to GAPDH mRNA values. The data represent the mean ± SEM of 5–6 mice. **P < 0.01 and *P < 0.05 (Tukey’s test).

Macrophages are attracted by a variety of chemokines, including CCL2, CCL3 and CCL4. We next examined the levels of CCL2, CCL3 and CCL4 mRNA in the damaged femurs on day 4. The levels of CCL3 mRNA were significantly elevated in the damaged femurs, compared with those in the contralateral intact femurs in *Plg* ^+/+^, *uPA* ^+/+^ and *tPA* ^+/+^ mice (Fig 6B). The elevations in CCL3 mRNA levels in the damaged femurs were reduced in *Plg* ^-/-^ and *uPA* ^-/-^ mice, compared with those in *Plg* ^+/+^ and *uPA* ^+/+^ mice, respectively (Fig 6B). On the other hand, there was no significant difference in CCL3 mRNA levels in the damaged femurs between *tPA* ^+/+^ and *tPA* ^-/-^ mice (Fig 6B). The levels of CCL2 and CCL4 mRNA were comparable between the damaged femurs and contralateral intact femurs in *Plg* ^+/+^, *uPA* ^+/+^ and *tPA* ^+/+^ mice (Fig 6A–6C). Plasminogen, uPA or tPA deficiency did not affect the levels of CCL2 and CCL4 mRNA in the damaged femurs (Fig 6A–6C).

### Effects of neutralizing anti-CCL3 antibody on the number of macrophages at the damaged site and decrease in the area of bone defect on day 4 in *uPA* ^+/+^ and *uPA* ^-/-^ mice

**Fig 7:**
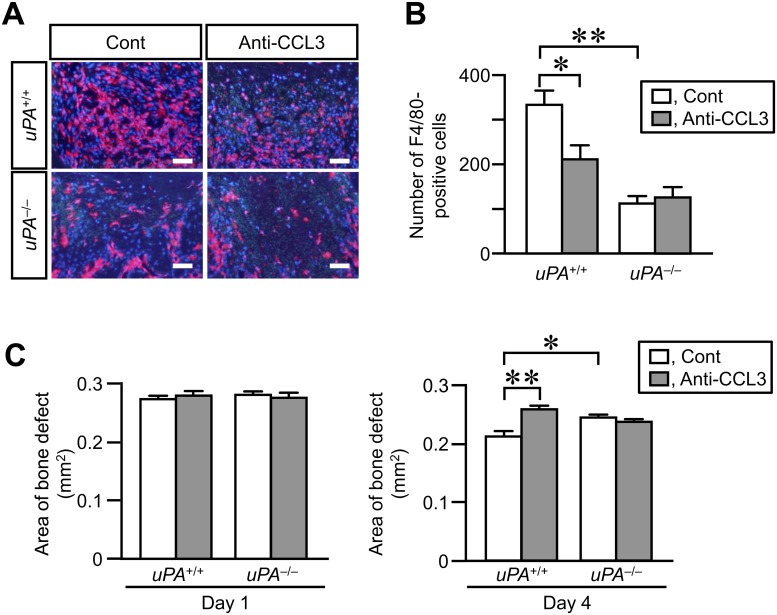
Effects of neutralizing anti-CCL3 antibody on the number of macrophages at the damaged site and the decrease in area of bone defect on day 4 in *uPA* ^+/+^ and uPA^-/-^ mice. (A) Microphotographs of immunostaining for F4/80 at the damaged site on day 4 after a femoral bone defect in *uPA* ^**+/+**^ and *uPA* ^**-/-**^ mice treated with normal IgG (Cont) or neutralizing anti-CCL3 antibody (Anti-CCL3). The results represent experiments performed on 5 mice in each group. Scale bars indicate 50 μm. (B) Quantification of the number of F4/80-positive cells per 0.1 mm^**2**^ in the microscopic fields in the damaged site on day 4 after a femoral bone defect in *uPA* ^**+/+**^ and *uPA* ^**-/-**^ mice treated with normal IgG or neutralizing anti-CCL3 antibody. The data represent the mean ± SEM of 5 mice. (C) Quantification of the bone defect area as assessed by qCT on days 1 and 4 in *uPA* ^**+/+**^ and *uPA* ^**-/-**^ mice treated with normal IgG or neutralizing anti-CCL3 antibody. The data represent the mean ± SEM from 5 mice. **P < 0.01 and *P < 0.05 (Tukey’s test).

We finally examined the role of CCL3 in the accumulation of macrophages at the damaged site of femur and the decrease in the area of bone defect in *uPA* ^+/+^ and *uPA* ^-/-^ mice. The number of macrophages at the damaged site of femur was significantly reduced by the administration of neutralizing anti-CCL3 antibody in *uPA* ^+/+^ mice on day 4 after femoral bone defect (Fig 7A and 7B). The decrease in the area of bone defect assessed by qCT was suppressed by the administration of neutralizing anti-CCL3 antibody in *uPA* ^+/+^ mice on day 4 (Fig 7C). Conversely, the administration of neutralizing anti-CCL3 antibody did not affect the number of macrophages at the damaged site of femur and the decrease in the area of bone defect in *uPA* ^-/-^ mice on day 4 (Fig 7A–7C).

## Discussion

Bone repair process is divided into three overlapping phases: inflammation, repair and remodeling [1]. The inflammatory cascade is initiated by cell and tissue damages. Then, neutrophils, macrophages and other inflammatory cells are recruited to the damaged site. The phase of repair begins with bone formation through intramembranous and endochondral ossification [1]. In the present study, uPA deficiency delayed bone repair and decreased the number of Osterix-positive cells at the damaged site of femur, coinciding with the decrease in the recruitment of macrophages, in the inflammation phase of the repair process. However, the number of ALP-positive cells as well as the degrees of bone and cartilage formation were similar in wild-type and uPA-deficient mice in the later phase of the bone repair process. Moreover, bone repair evaluated by qCT was comparable between wild-type and uPA-deficient mice in the later phase of the bone repair process as we previously reported [29]. uPA therefore might play primary roles in the phase of inflammation during bone repair.

Macrophages play important roles in the repair process through stimulation of angiogenesis, engulfment of cellular debris and efferocytosis of apoptotic cells in many tissues [4]. As for bone repair, a previous study showed that preventing the recruitment of inflammatory macrophages in a mouse non-union fracture model resulted in reduced endochondral ossification together with impaired vascularization [33]. Moreover, Alexander *et al*. showed that depletion of macrophages including osteomacs, a specialized resident bone macrophage population, suppresses new bone formation through intramembranous ossification and mineralization in a mouse tibial injury model [34]. These findings suggest that macrophages recruited to the site of injury have a positive influence on the process of bone repair. In the present study, we revealed that uPA-deficiency and plasminogen-deficiency decreased the numbers of recruited macrophages at the damaged site, suggesting that uPA and plasminogen contribute to the recruitment of macrophages to the site of bone injury. Moreover, our present and previous studies indicate that uPA deficiency and plasminogen deficiency delay bone repair in the phase of inflammation during the repair process [18]. These findings suggest that uPA and plasminogen are necessary for early bone repair process (the inflammation phase), including the recruitment of macrophages to the damaged site.

Macrophages are attracted to the damaged site by chemoattractants, including the CC chemokines, CCL2, CCL3 and CCL4, after tissue injury [1–3]. CCL2 is responsible for monocyte trafficking in the body. A previous study revealed that deficiency of CCR2, the receptor for CCL2, reduces the infiltration of macrophages to the fracture site in mice [33]. The levels of CCL2 mRNA were elevated in the injured mouse tibia on day 2 in the previous study [33]. These findings suggested that CCL2 might be involved in bone repair through regulation of macrophage recruitment to the sites of bone injury. In the present study, we showed that the levels of CCL3, but not CCL2 and CCL4, mRNA were elevated in the damaged femurs of wild-type mice on day 4 after a bone defect. Moreover, the neutralization of CCL3 partially suppressed the recruitment of macrophages to the damaged site of femur and delayed bone repair on day 4 after bone defect. These results suggest that CCL3 is involved in bone repair by recruiting macrophages to the damaged site after bone injury.

CCL3 is expressed by immune cells, such as macrophages, lymphocytes, mast cells and natural killer cells [3]. Osteoblasts also produce CCL3 upon stimulation by TNF-α, IL-1α and IL-1β [35,36]. The increase in CCL3 levels contributes to the development of bone disease in multiple myeloma by supporting tumor growth and regulating osteoclast differentiation [37]. Moreover, a previous study suggested that CCL3 reduces bone formation by inhibiting osteoblast function, including mineralization and osteocalcin production in myeloma-induced bone disease [38]. Taddei *et al*. revealed that CCL3 deficiency decreased the mechanical loading-induced bone resorption in mice, suggesting an involvement of CCL3 in bone remodeling [39]. Our present data indicated that CCL3 is involved in bone repair through enhancement of macrophage recruitment to the damaged site after a femoral bone defect. We can therefore speculate that the effects of CCL3 on macrophage recruitment rather than its direct effects on osteoblasts and osteoclasts are relevant to the process of bone repair during the inflammation phase.

We revealed that uPA deficiency and plasminogen deficiency suppressed the elevations in CCL3 mRNA levels in the damaged femurs in the present study. Moreover, the neutralization of CCL3 did not further suppress the decrease in macrophage recruitment to the damaged sites in uPA-deficient mice. These results suggest that CCL3 is involved in uPA-dependent macrophage recruitment to the site of bone injury. Previous studies indicate that IL-13 suppresses CCL3 expression induced by stimulation with proinflammatory cytokines [40,41]. In the present study, the levels of IL-13 mRNA are elevated in the damaged femur of uPA-deficient mice, but not in wild-type mice. We therefore speculated that uPA might contribute to the regulation of CCL3 levels through IL-13 expression in the damaged femur during bone repair.

Macrophages recruited to the site of tissue injury show functional phenotypes regulated by the balance of cytokines, including IFN-γ, TNF-α, IL-4 and IL-13, in the tissue microenvironment [4,10,42]. We revealed that macrophage phagocytosis was reduced in uPA-deficient and plasminogen-deficient mice at the damaged site, suggesting that uPA or plasminogen deficiency suppresses the activation of the recruited macrophages at the damaged site of the femur. We also showed that uPA or plasminogen deficiency did not affect the levels of cytokine mRNA, including TNF-α, IL-1β, IL-6, IL-4 and IFN-γ in the damaged femur. These results suggest that the balance of cytokines at the damaged site of the femur might not be responsible for the decrease in the activation of the recruited macrophages. However, our data indicate that the levels of IL-13 and IL-10 mRNA are elevated in the damaged femur of uPA-deficient and plasminogen-deficient mice, but not in that of their wild-type mice. IL-13 and IL-10, anti-inflammatory mediators, regulate macrophage functions [4,43,44]. We therefore cannot rule out the possibility that IL-13 and/or IL-10 might affect the activation of recruited macrophages at the damaged site of the femur in uPA-deficient and plasminogen-deficient mice. Further studies are necessary to clarify the details in mechanisms of uPA and plasminogen-dependent macrophage activation at the damaged site during bone repair.

Plasminogen activators, uPA and tPA, expressed in osteoblasts, are enhanced by several stimuli with hormones and factors [11–13]. We recently reported that tPA deficiency delayed bone repair and decreased the proliferation of osteoblasts at the damaged site during bone repair process in mice [29]. Moreover, the proliferation of osteoblasts from tPA-deficient mice was less than that from wild-type mice [29]. These findings suggested that the effects of tPA on bone repair might be due to a cell autonomous effect on proliferation in osteoblasts *in vivo*. Moreover, our previous study suggested that the effects of tPA on bone repair might be fibrinolytic system-independent, which might explain the differences in macrophage accumulation between uPA-deficient and tPA-deficient mice in the present study. In our study, uPA deficiency delayed the inflammation phase of bone repair in mice, whereas it did not affect the levels of osteogenic genes, including Runx2, Osterix, ALP and type I collagen mRNA, ALP activity and mineralization in primary osteoblasts. The effects of uPA on bone repair, therefore, might be due to a non-cell autonomous effects in osteoblasts *in vivo*. We previously reported that plasminogen deficiency impairs the formation of bone tissues at the damaged sites during bone repair in mice [18]. On the other hand, plasminogen deficiency enhanced mineralization and the levels of osteogenic genes in primary osteoblasts, suggesting that plasminogen suppresses the mineralization and differentiation of osteoblasts [18]. The effect of plasminogen on bone repair, therefore, might be due to a non-cell autonomous effect in osteoblasts *in vivo*, but not its direct effects on osteoblasts. Moreover, the effects of uPA deficiency on macrophage recruitment as well as vessel formation related to VEGF and TGF-β at the damaged site in the inflammation phase during bone repair in the present study were similar to those of plasminogen deficiency in our previous study [18]. Taken together, these results suggest that the effects of uPA on the bone repair process *in vivo* is dependent of tissue fibrinolytic system. Further studies using uPA and tPA double-deficient mice as well as compound heterozygous mice might be useful to clarify the details in mechanisms of tissue fibrinolytic system-related bone repair process.

In conclusion, our results provide novel evidence that the uPA/plasminogen system contributes to macrophage recruitment through CCL3 at the site of bone injury and the activation of the recruited macrophages in mice, thereby leading to the enhancement of the repair process. The regulation of the tissue fibrinolytic system may lead to new approaches to improve bone repair and facture healing.

## Supporting Information

S1 Fig Hematoxylin and eosin-stained vertical sections collected from the damaged site in uPA+/+ and *uPA* ^-/^- mice on day 7 after femoral bone defect. Scale bars indicate 50 μm. The results represent experiments performed on 5 mice in each group.
(TIF)

S2 Fig Effects of uPA deficiency on chondrogenesis. (A) Quantification of Alcian blue-positive area at the damaged site in *uPA* ^+/+^ and *uPA* ^-/-^ mice on day 7 after femoral bone defect. The data represent the mean ± SEM of 5 mice. (B) Quantification of the area of the metachromatic region in sections stained with toluidine blue in *uPA* ^+/+^ and *uPA* ^-/-^ mice at the damaged site on day 7 after femoral bone defect. The data represent the mean ± SEM of 5 mice. (C) Relative mRNA levels of aggrecan, type II collagen (Col II) and type X collagen (Col X) in the damaged femur of *uPA* ^+/+^ and *uPA* ^-/-^ mice on day 7. The data represent the mean ± SEM of 6 mice.
(TIF)

S3 Fig Effects of uPA deficiency on the levels of osteogenic genes and mineralization in primary osteoblasts. (A) Relative mRNA levels of Runx2, Osterix, ALP and type I collagen (Col I) in calvarial osteoblasts derived from *uPA* ^+/+^ and *uPA* ^-/-^ mice. (B) Quantification of ALP activity in calvarial osteoblasts derived from *uPA* ^+/+^ and *uPA* ^-/-^ mice. (C) Mineralization of calvarial osteoblasts from *uPA* ^+/+^ and *uPA* ^-/-^ mice. Images of Alizarin red-stained cells (C, left) and quantification of Alizarin red staining by measurement of the absorbance of the extracted stain (C, right). The data represent the mean ± SEM of 3 (A, C) and 5 (B) experiments.
(TIF)

S4 Fig Effects of uPA deficiency on vessel formation at the damaged site on day 7. (A) Microphotographs of CD31 immunostaining at the damaged site on day 7 after femoral bone defect in *uPA* ^+/+^ and *uPA* ^-/-^ mice. The results represent experiments performed in 5 mice of each group. Scale bars indicate 50 μm. (B) Quantification of blood vessels at the damaged site on day 7 in *uPA* ^+/+^ and *uPA* ^-/-^ mice. The number and total luminal area of blood vessels per 0.1 mm^2^ of the microscopic fields in the damaged site. The data represent the mean ± SEM of 5 mice.
(TIF)

S1 Table Primers used for real-time PCR experiments. (DOC)
