# Supplementary figures and images for: The Tissue Fibrinolytic System Contributes to the Induction of Macrophage Function and CCL3 during Bone Repair in Mice
Source: PLoS One. 2015 Apr 20;10(4):e0123982. doi: 10.1371/journal.pone.0123982 (PMC4404328; doi:10.1371/journal.pone.0123982)

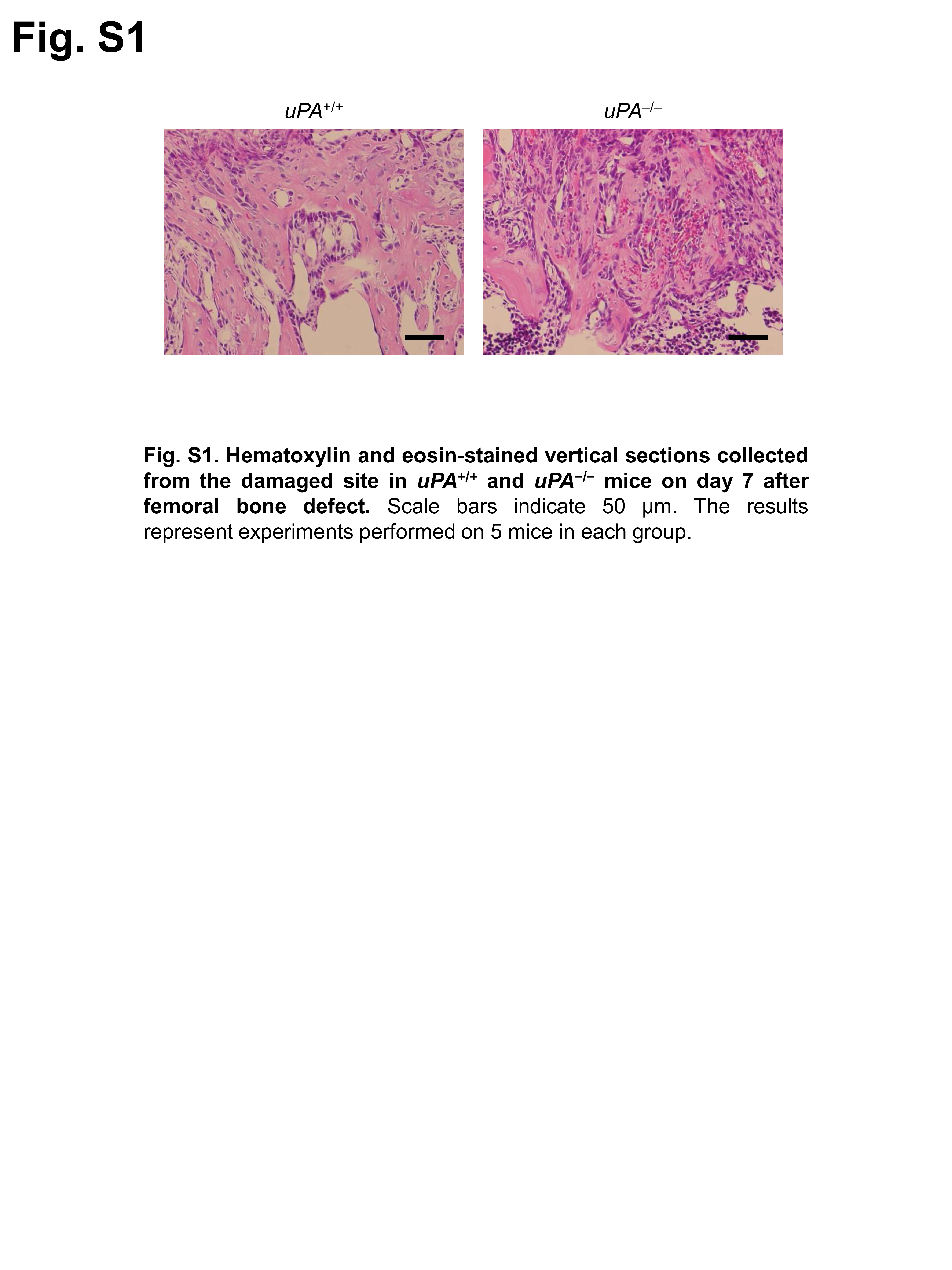

Supplement: S1 Fig — The results represent experiments performed on 5 mice in each group. (TIF) [file pone.0123982.s001.tif]

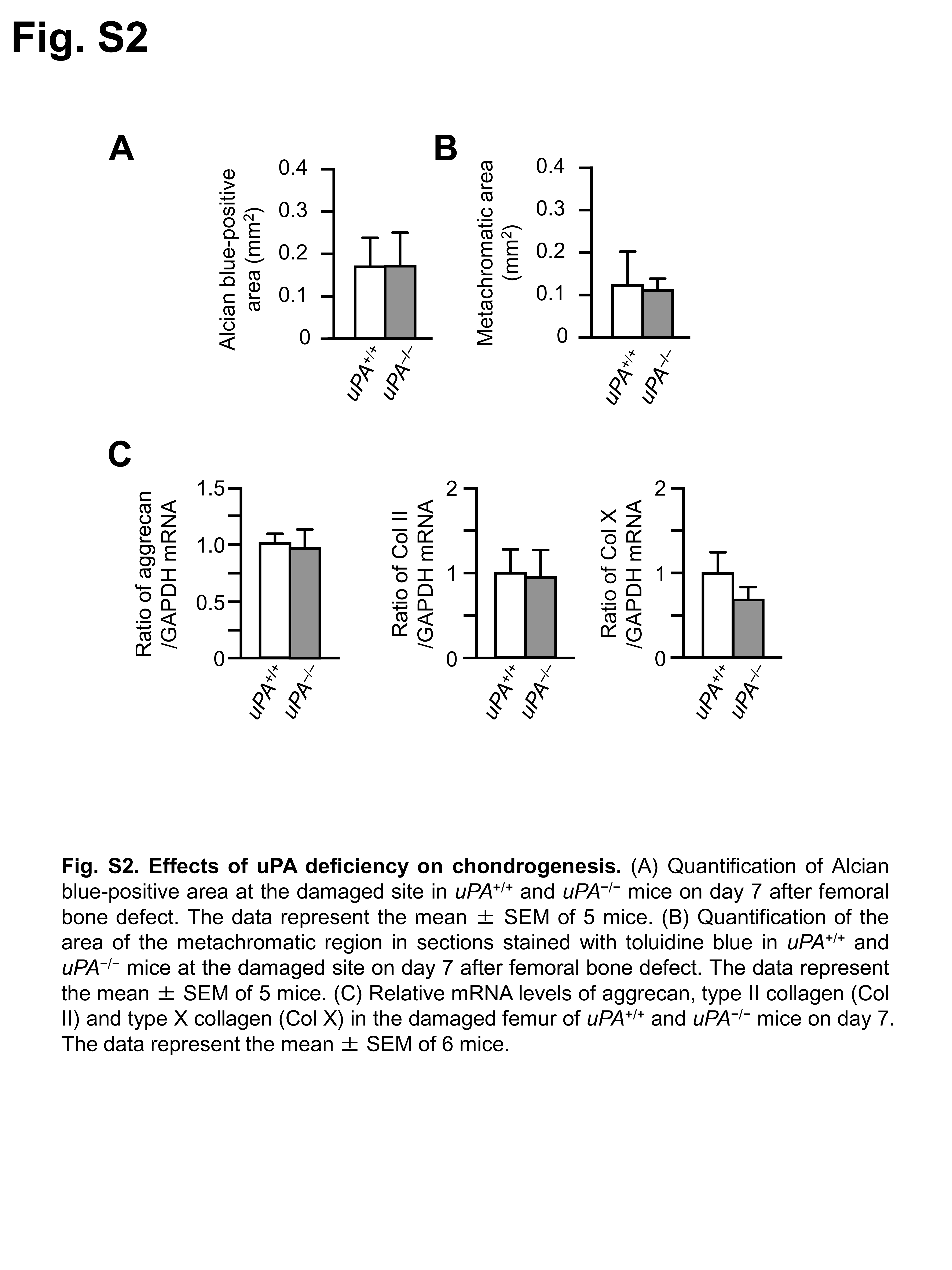

Supplement: S2 Fig — (A) Quantification of Alcian blue-positive area at the damaged site in uPA +/+ and uPA -/- mice on day 7 after femoral bone defect. The data represent the mean ± SEM of 5 mice. (B) Quantification of the area of the metachromatic region in sections stained with toluidine blue in uPA +/+ and uPA -/- mice at the damaged site on day 7 after femoral bone defect. The data represent the mean ± SEM of 5 mice. (C) Relative mRNA levels of aggrecan, type II collagen (Col II) and type X collagen (Col X) in the damaged femur of uPA +/+ and uPA -/- mice on day 7. The data represent the mean ± SEM of 6 mice. (TIF) [file pone.0123982.s002.tif]

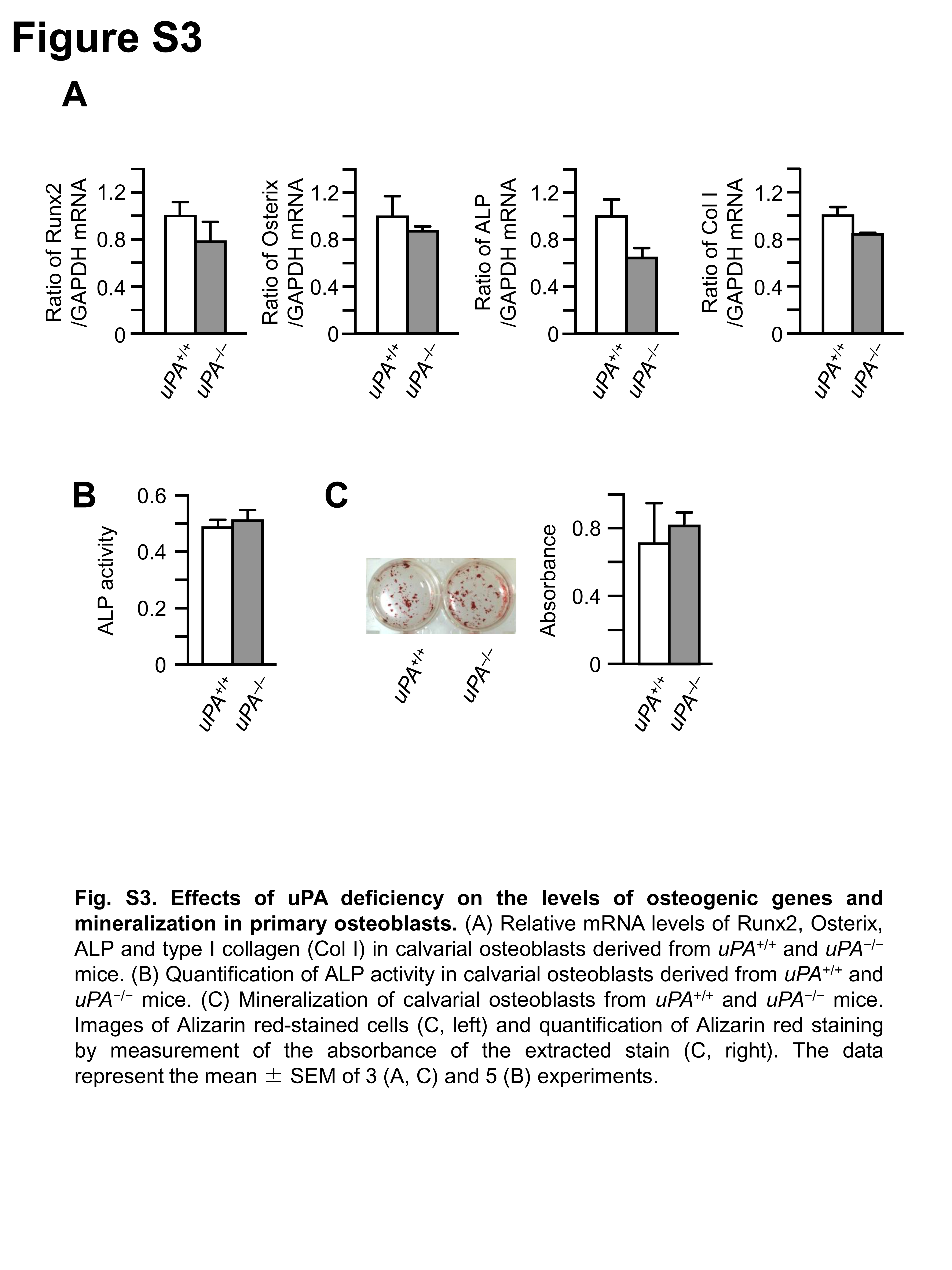

Supplement: S3 Fig — (A) Relative mRNA levels of Runx2, Osterix, ALP and type I collagen (Col I) in calvarial osteoblasts derived from uPA +/+ and uPA -/- mice. (B) Quantification of ALP activity in calvarial osteoblasts derived from uPA +/+ and uPA -/- mice. (C) Mineralization of calvarial osteoblasts from uPA +/+ and uPA -/- mice. Images of Alizarin red-stained cells (C, left) and quantification of Alizarin red staining by measurement of the absorbance of the extracted stain (C, right). The data represent the mean ± SEM of 3 (A, C) and 5 (B) experiments. (TIF) [file pone.0123982.s003.tif]

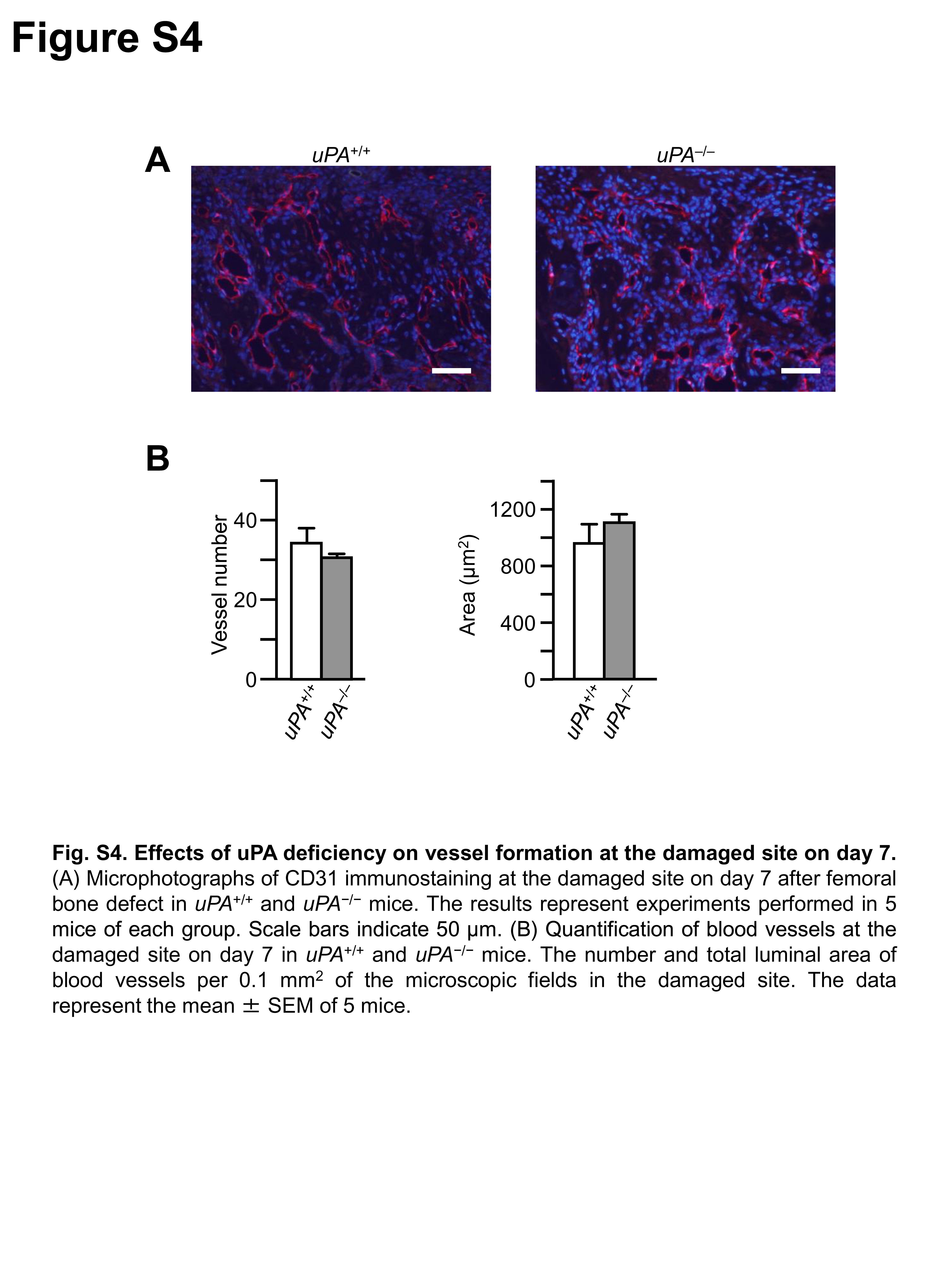

Supplement: S4 Fig — (A) Microphotographs of CD31 immunostaining at the damaged site on day 7 after femoral bone defect in uPA +/+ and uPA -/- mice. The results represent experiments performed in 5 mice of each group. Scale bars indicate 50 μm. (B) Quantification of blood vessels at the damaged site on day 7 in uPA +/+ and uPA -/- mice. The number and total luminal area of blood vessels per 0.1 mm2 of the microscopic fields in the damaged site. The data represent the mean ± SEM of 5 mice. (TIF) [file pone.0123982.s004.tif]
