# Supplementary material for: The Tissue Fibrinolytic System Contributes to the Induction of Macrophage Function and CCL3 during Bone Repair in Mice
Source: PLoS One. 2015 Apr 20;10(4):e0123982. doi: 10.1371/journal.pone.0123982 (PMC4404328; doi:10.1371/journal.pone.0123982)
Supplement: S1 Table — (DOC) [file pone.0123982.s005.doc]

Supplementary Table 1. Primers used for real-time PCR experiments.

Gene Primer sequence

VEGF Forward 5’-CTGTGCAGGCTGCTGTAACG-3’

Reverse 5’- GTTCCCGAAACCCTGAGGAG-3’

TGF-β1 Forward 5’-CCTCTGTCACCTGCTCAACA-3’

Reverse 5’-GATGAATTGGCGTGGAATCT-3’

BMP-2 Forward 5’-GGTCACAGATAAGGCCATTGC-3’

Reverse 5’-GCTTCCGCTGTTTGTGTTTG-3’

TNF-α Forward 5’-CCCAGACCCTCACACTCAGATC-3’

Reverse 5’-GCCACTCCAGCTGCTCCTC-3’

IL-1β Forward 5’-GGTCAAAGGTTTGGAAGCAG-3’

Reverse 5’-TGTGAAATGCCACCTTTTGA-3’

IL-6 Forward 5’-GTTCTCTGGGAAATCGTGGA-3’

Reverse 5’-GGAAATTCGGGGTAGGAAGGA-3’

IL-4 Forward 5’-ACAGGAGAAGGGACGCCAT-3’

Reverse 5’-GAAGCCCTACAGACGAGCTCA-3’

IL-13 Forward 5’-**GGATATTGCATGGCCTCTGTAAC**-3’

Reverse 5’-**AACAGTTGCTTTGTGTAGCTGA**-3’

IL-10 Forward 5’-**GCTCTTACTGACTGGCATGAG**-3’

Reverse 5’-CGCAGCTCTAGGAGCATGTG-3’

IFN-γ Forward 5’-GCATAGATGTGGAAGAAAAGAGTCTCT-3’

Reverse 5’-TGGCTCTGCAGGATTTTCATG-3’

CCL2 Forward 5’-CCACTCACCTGCTGCTACTCA-3’

Reverse 5’-TGGTGATCCTCTTGTAGCTCTCC-3’

CCL3 Forward 5’-**CCTCTGTCACCTGCTCAACA**-3’

Reverse 5’-**GATGAATTGGCGTGGAATCT**-3’

CCL4 Forward 5’-**CCCACTTCCTGCTGTTTCTC**-3’

Reverse 5’-**GAGGAGGCCTCTCCTGAAGT**-3’

Aggrecan Forward 5’-CCTGCTACTTCATCGACCCC-3’

Reverse 5’-AGATGCTGTTGACTCGAACCT-3’

Col II Forward 5’-CCTCCGTCTACTGTCCACTGA-3’

Reverse 5’-ATTGGAGCCCTGGATGAGCA-3’

Col X Forward 5’-TGGGTAGGCCTGTATAAAGAACGG-3’

Reverse 5’-CATGGGAGCCACTAGGAATCCTGAGA-3’

Runx2 Forward 5’-AAATGCCTCCGCTGTTATGAA-3’

Reverse 5’-GCTCCGGCCCACAAATCT-3’

Osterix Forward 5’-AGCGACCACTTGAGCAAACAT-3’

Reverse 5’-GCGGCTGATTGGCTTCTTCT-3’

ALP Forward 5’-ATCTTTGGTCTGGCTCCCATG-3’

Reverse 5’-TTTCCCGTTCACCGTCCAC-3’

Col I Forward 5’-GGTCAAAGGTTTGGAAGCAG-3’

Reverse 5’-TGTGAAATGCCACCTTTTGA-3’

GAPDH Forward 5’-AGGTCGGTGTGAACGGATTTG-3’

Reverse 5’-GGGGTCGTTGATGGCAACA-3’

VEGF, vascular endothelial growth factor; TGF-β1, transforming growth factor-β1; BMP-2, bone morphogenetic protein-2; TNF-α, tumor necrosis factor-α; IL, interleukin; IFN-γ, interferon-γ; Col II, type II collagen; Col X, type X collagen; ALP, alkaline phosphatase; Col I, type I collagen; GAPDH, glyceraldehyde-3-phosphate dehydrogenase.
